# Younger patients with chronic lymphocytic leukemia benefit from rituximab treatment: A single center study in China

**DOI:** 10.3892/ol.2013.1177

**Published:** 2013-02-05

**Authors:** ZHENSHU XU, JINYAN ZHANG, SHUNQUAN WU, ZHIHONG ZHENG, ZHIZHE CHEN, RONG ZHAN

**Affiliations:** Department of Hematology, Union Hospital of Fujian Medical University, Fuzhou, Fujian 350001, P.R. China

**Keywords:** chronic lymphocytic leukemia, immunoglobulin variable heavy-chain, immunophenotype, chemoimmunotherapy, rituximab

## Abstract

Chronic lymphocytic leukemia (CLL) is characterized by high heterogeneity in clinical features and outcomes in Western countries and China. In this study, the clinical and laboratory data of 210 CLL patients who were admitted to a single center in China between 2002 and 2011 were retrospectively analyzed. CLL patients had a median age of 60.2 years (range, 35–92 years) and CLL occurred more often in elderly female patients than in male patients (female:male, 1.2:1). The overall response rate [ORR, complete remission (CR) + partial remission (PR)] in the entire cohort of patients was 69.5% and the median overall survival (OS) was 67 months (95% CI, 57.88–76.11). In univariate analysis, an age of >60 years, chromosome 17p deletion (17p^−^) and elevated β2-MG were associated with a worse OS. Patients with all three poor prognostic factors had a worse outcome than patients with only one or two factors. Patients with 17p^−^ had a significantly lower ORR (P=0.008) and shorter OS (P=0.001) than those without 17p^−^. Rituximab (R)-based treatment was able to overcome the poor prognosis associated with 17p^−^. Moreover, the addition of R to fludarabine (F) and cyclophosphamide (C) treatment significantly improved the OS (P=0.012) compared with FC alone in younger patients. However, there were no significant benefits for older patients (P=0.07). This implies that the age of a patient is important in their response to therapy and survival.

## Introduction

Although chronic lymphocytic leukemia (CLL) patients with early-stage disease have a life expectancy of >10 years, those with progression or who have advanced-stage disease (Binet stages B and C) have a median survival of 2–7 years (1). A number of patients are asymptomatic and may survive for decades without requiring treatment, whereas others experience an aggressive form of disease and may succumb to the disease or due to therapy-associated complications shortly after diagnosis. However, the majority of research on CLL is from Western countries, with few studies reporting results in Chinese patients.

For more than 30 years, CLL has been treated with various chemotherapeutic drugs, including the alkylating drug chlorambucil (Ch). In the past decade, the combination of purine analogs with alkylating drugs, particularly fludarabine (F) and cyclophosphamide (C), has improved the rate of clinical response, complete remission (CR) and progression-free survival (2). Additionally, the use of rituximab (R) has improved the efficacy of CLL treatment, with complete response rates of 40–70% (3–5). Since these trials enrolled younger patients with a good performance status and no severe morbidities, the results of these studies are most relevant to the treatment of younger Western patients with CLL. The effect of anti-CD20 antibodies in Asian patients with CLL remains to be determined. The primary objective of our study was to retrospectively analyze the clinical characteristics of CLL patients in China and to evaluate the outcome of R treatment.

## Patients and methods

### Patients

Between January 2002 and December 2011, 210 patients with CLL were treated in our institution. The history, physical findings and laboratory data of the patients were collected for analysis. The diagnostic criteria for CLL were as follows: i) Persistent lymphocytosis of >5×10^9^/l lasting for ≥3 months; ii) morphologically small- to medium-sized lymphocytes without nucleoli; iii) no cell membrane hair formation; iv) CD5^+^, CD19^+^ and CD10^−^ immunophenotypes; and v) no cyclin D1 expression. The study methods were reviewed and approved by Union Hospital of Fujian Medical University Review Board.

### Serum parameters

Serum and heparinized blood samples were obtained from all patients before treatment. Serum lactate dehydrogenase (LDH) and serum β2-microglobulin (β2-MG) levels were evaluated by radioimmunoassay.

### Immunoglobulin variable heavy-chain (IgVH) mutation status analysis

To determine the VH gene mutation status, reverse transcription-polymerase chain reaction (RT-PCR) was performed using VH primers (6). Amplified PCR products were purified and directly sequenced using a sequencing kit (Applied Biosystems, Carlsbad, CA, USA). Mutation status was determined by comparison with the consensus germline sequence according to IgBlast (www.ncbi.nlm.nih.gov/igblast) and IMGT/V-QUEST (http://www.imgt.org). We determined the gene as ‘mutated’ when the sequence deviated by ≥2% from the consensus sequence.

### Evaluation of CD38 and ZAP-70 expression

Flow cytometry analysis in this study was performed on a FACSCalibur flow cytometer (BD Biosciences, Franklin Lakes, NJ, USA). The expression of CD38 was analyzed by 3-color immunofluorescence (7) and the detection of ZAP-70 was performed according to previously reported methods (8). A cut-off point of 30% positive cells was selected to discriminate CD38^−^ from CD38^+^ CLL. A cut-off point of 20% was used to distinguish ZAP-70^−^ from ZAP-70^+^ CLL.

### Cytogenetic analysis

Interphase fluorescence *in situ* hybridization (FISH) was carried out for the detection of trisomy 12 and chromosome deletions at 13q14.3, 11q22.3 and 17p13.1 loci. A chromosome 12-specific α-satellite probe was used to identify trisomy 12. For the detection of a 13q14.3 deletion, a locus-specific probe (LSI D13D25) was cohybridized with the 13q34 telomeric probe as an internal control for nullisomy. Dual-color hybridizations using the appropriate centromere-specific probes and unique sequence-specific probes for the ATM (LSI ATM) and TP53 (LSI P53) loci were performed for the detection of 11q22.3 and 17p13.1 deletions, respectively. All probes were purchased from Vysis (Abbott, Shanghai, China) and FISH procedures were performed according to the manufacturer’s instructions. For each hybridization, ≥200 interphase nuclei were assessed. Patients were categorized into low- (13q14.3 deletion and normal), intermediate- (trisomy 12) and high- (11q22.3 and 17p13.1 deletions) risk groups for subsequent analysis.

### Treatments

Treatment consisted of six 28-day courses of intravenous F at 25 mg/m^2^ per day and C at 250 mg/m^2^ per day for the first three days of each treatment course, with or without R at a dose of 375 mg/m^2^ on day 0 of each course. Ch was initially administered orally at 0.4 mg/kg body weight (BW) on day one and was increased by 0.1 mg/kg for each treatment course up to 0.8 mg/kg BW if treatment was well tolerated. Maintenance therapy was performed with two cycles of the original treatment course after achieving CR.

### Statistical methods

Clinical data are presented using descriptive statistics. The χ^2^ test was used to compare clinical characteristics between groups. Overall survival (OS) was defined as the time between the date of diagnosis and the date of the last follow-up or death due to any cause. For univariate survival analysis, the Kaplan-Meier method for incomplete observation was used. The estimated survival curves were compared using the log-rank test. A multivariate analysis of the potential factors affecting the OS was performed using a step-wise Cox proportional-hazard regression method. P<0.05 was considered to indicate a statistically significant difference. All tests were two-tailed with a multiple significance level of α=5%.

## Results

### Patients

**Figure 1:**
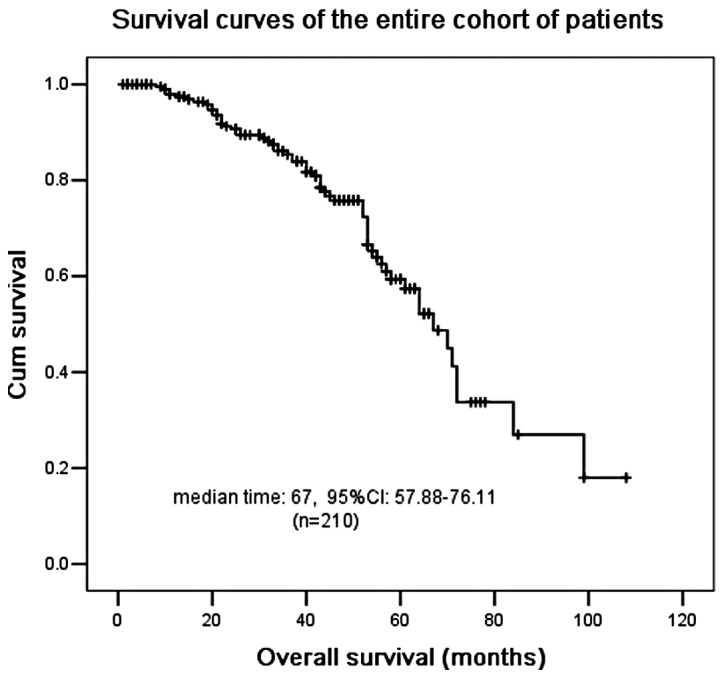
Survival curve for the entire cohort of patients with CLL (n=210; median survival time is 67 months; 95% CI, 57.88–76.11). CLL, chronic lymphocytic leukemia.

Between January 2002 and December 2011, 210 patients with CLL from a single institution in China were enrolled in this study and followed up for survival. The main clinical characteristics of the 210 patients in this study are shown in Table I. The median follow-up for the entire group was 68 months (range, 4–110 months). There were 95 males and 115 females in this study, whose age at the time of enrollment ranged from 35*–*92 years with a median age of 60.2 years (Fig. 1). Immunophenotypic data, available for 202 of the 210 patients, showed that all cases of leukemia were CD19^+^, 196/202 were CD5^+^, 188/202 were CD20^+^ [61/188 expressed (+) and 127/188 expressed (++)] and 200/202 were CD23^+^. All cases were confirmed to be of the B-cell type. At the time of enrollment, 53 patients had stage A, 120 had stage B and 37 had stage C disease according to the Binet system.

### Treatment and associated adverse events

The overall response rates [ORR, CR + partial remission (PR)] were significantly different among the treatment regimens (P<0.0001) and the lowest ORR of 44% was identified in patients treated with Ch (95% CI, 0.30–0.48). The ORR was 68.5% in patients treated with F and C (95% CI, 0.60–0.72) and this further improved in patients treated with F, C and R (77.8%; 95% CI, 0.70–0.92). There was no significant difference in ORRs between patients ≤60 years old and patients >60 years old (P=0.112).

The most frequently occurring adverse events for R- or F-based treatments in this study were fatigue, hypersensitive skin reactions, hematological toxicity and GI events (nausea, vomiting and diarrhea). There were ten reports of tumor lysis syndrome, all in patients who had received their first cycle of chemotherapy. Neutropenia was observed in 25% of patients, including grade 3 or 4 in 14% of patients, and thrombocytopenia was observed in 9% of patients. During maintenance therapy, 54% of patients had low Ig serum levels and 47 patients experienced grade 3 or 4 infectious episodes, including 26 patients with pneumonia, 10 with appendicitis, 6 cases of myositis and 5 of herpes zoster. The hepatitis B virus was activated in 10% (21 cases) of patients after R-based treatment. Severe infections were of particular interest since they are a major cause of morbidity and mortality in CLL patients. Notably, there were 39 patients treated with F, C and R who experienced grade 3 and 4 infections, however, only eight patients who received other treatments (including Ch) had grade 3 and 4 infections.

### Survival analysis

**Figure 2:**
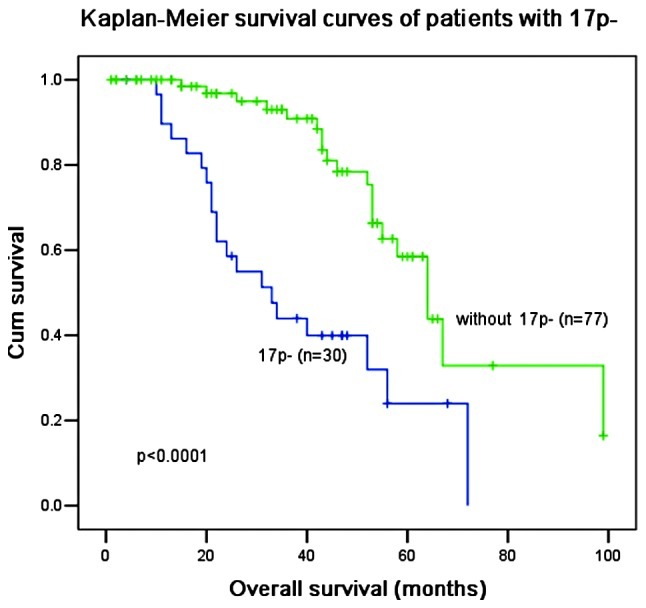
Kaplan-Meier survival curves show the impact of the biological feature of 17p^−^ on the overall survival (OS) of patients with CLL. Patients with 17p^−^ have a significantly shorter OS than those without 17p^−^ (P<0.0001, log-rank test). CLL, chronic lymphocytic leukemia; 17p^−^, chromosome 17p deletion.

**Figure 3:**
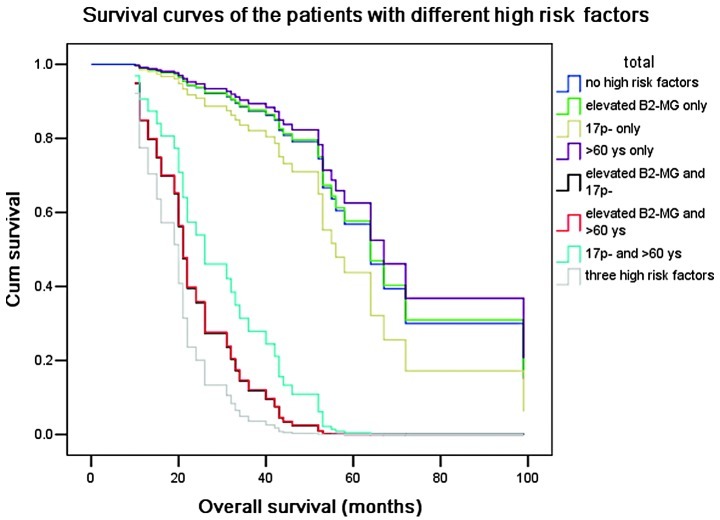
Kaplan-Meier survival curves show the impact of various high-risk factors on the OS in cohort patients with CLL. OS, overall survival; CLL, chronic lymphocytic leukemia.

The median OS for the entire cohort was 67 months (Fig. 1). Univariate analysis showed that an age of >60 years old, chromosome 17p deletion (17p^−^) and elevated β2-MG levels were associated with a worse overall survival. The patients who harbored 17p^−^ had a significantly lower ORR (40 vs. 72%; P=0.008; Table I) and a shorter OS (33 vs. 64 months; P<0.0001; Fig. 2) than patients without 17p^−^. Patients with all three poor prognostic factors had a worse median OS than patients with only one or two factors (21–28 months vs. 54–62 months; P=0.02; Fig. 3).

**Figure 4:**
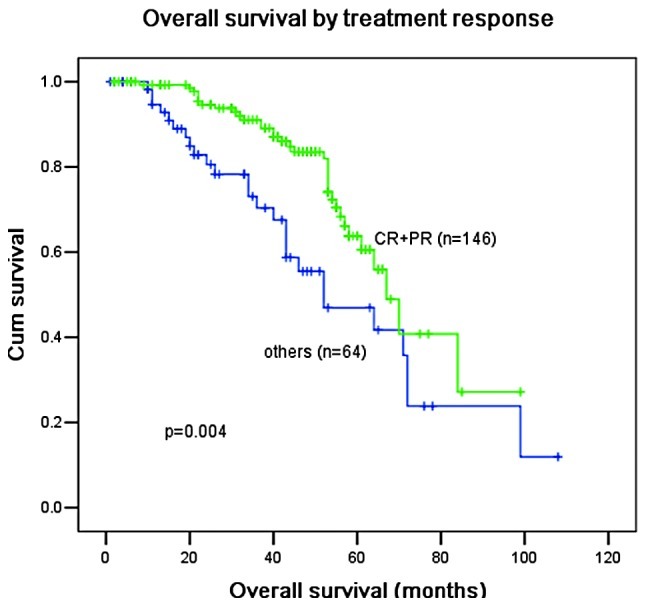
Overall survival by treatment response. CR, complete remission; PR, partial remission.

**Figure 5:**
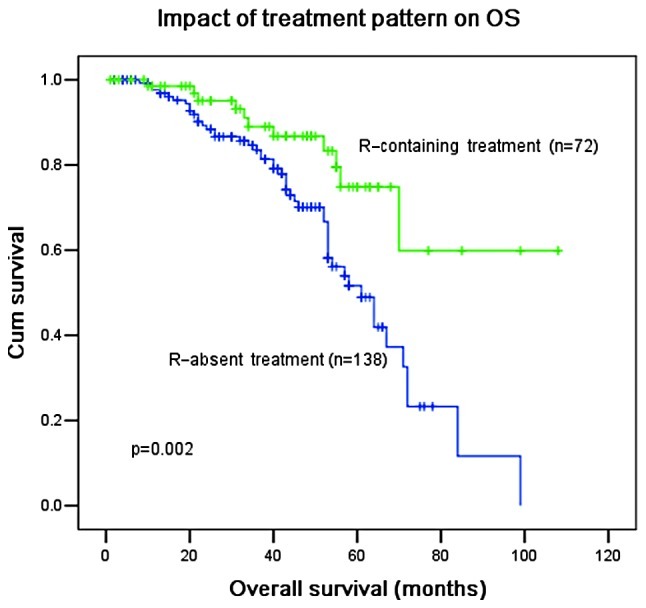
Impact of treatment pattern on the OS. The Kaplan-Meier survival curves show the impact of treatment pattern on the OS. The patients who received rituximab (R)-containing treatment have a significantly better OS than those who did not (P=0.002, log-rank test). OS, overall survival.

Patients who achieved CR or PR after four cycles of therapy had a better OS than patients who failed to achieve CR or PR (Fig. 4). The OS of patients was significantly improved with R-based treatment (P=0.002; Fig. 5).

**Figure 6:**
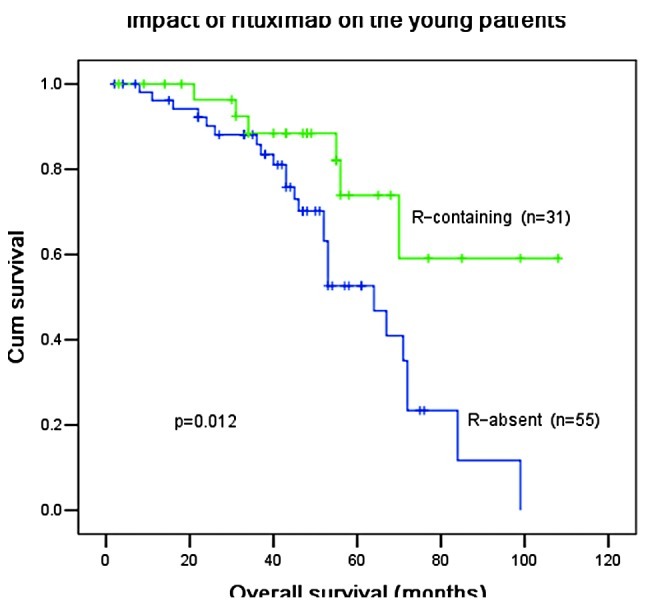
Kaplan-Meier survival curves show the impact of rituximab (R)-containing treatment on the OS of young patients (≤60 years old) with CLL. Patients with R treatment have a significantly longer OS than those without R treatment (P= 0.012, log-rank test). OS, overall survival; CLL, chronic lymphocytic leukemia.

**Figure 7:**
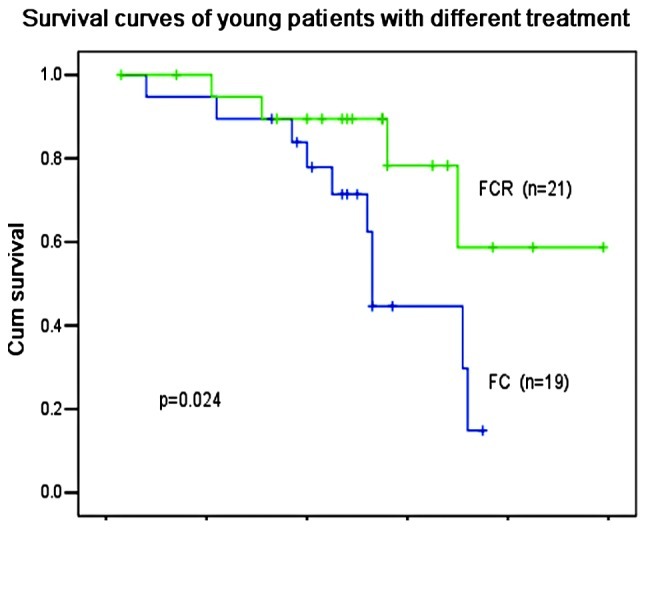
Kaplan-Meier survival curves show the impact of the two treatments (FCR and FC) on the OS of young patients (≤60 years old) with CLL. FCR, fludarabine, cyclophosphamide and rituximab; FC, fludarabine and cyclophosphamide; OS, overall survival; CLL, chronic lymphocytic leukemia.

**Figure 8:**
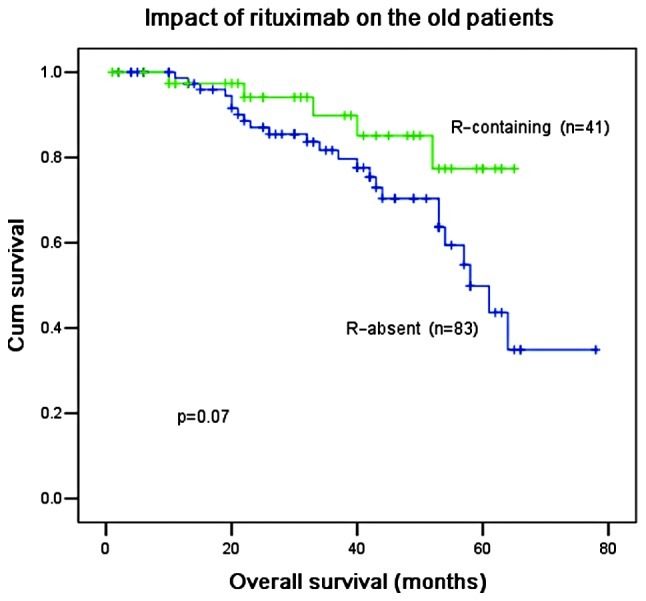
Kaplan-Meier survival curves show the impact of rituximab (R)-containing treatment on the OS of older patients (>60 years old) with CLL. Patients with R treatment have a longer OS than those without R treatment (P=0.07, log-rank test). OS, overall survival; CLL, chronic lymphocytic leukemia.

**Figure 9:**
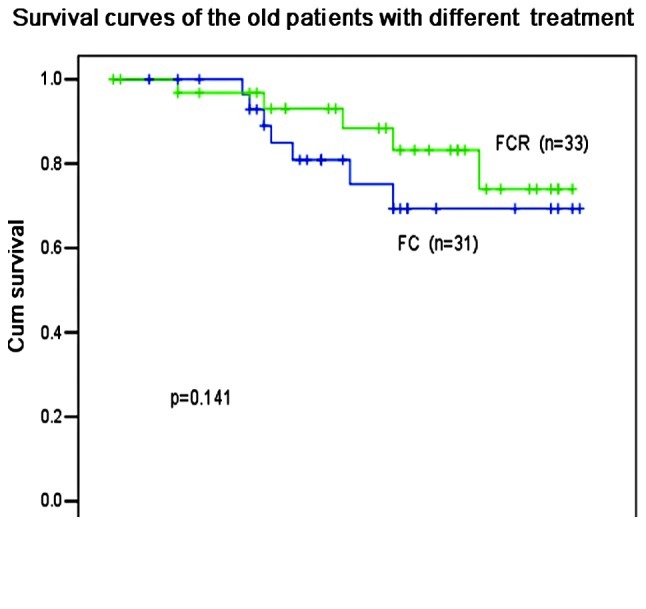
Kaplan-Meier survival curves show the impact of the two treatments (FCR and FC) on the OS of the older patients (>60 years old) with CLL. FCR, fludarabine, cyclophosphamide and rituximab; FC, fludarabine and cyclophosphamide; OS, overall survival; CLL, chronic lymphocytic leukemia.

**Figure 10:**
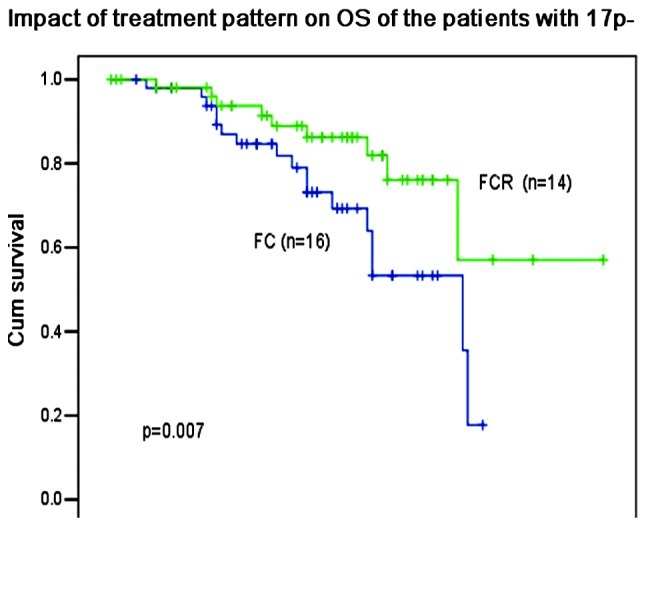
Impact of the treatment pattern on the OS of patients with 17p^−^. OS, overall survival; 17p^−^, chromosome 17p deletion; FCR, fludarabine, cyclophosphamide and rituximab; FC, fludarabine and cyclophosphamide.

In patients ≤60 years old, R-based treatment significantly improved the OS compared with treatment without R (P=0.012; Fig. 6). R treatment also increased the OS compared with F and C treatment (P=0.024; Fig. 7), although F and C treatment produced a higher (not significant) ORR than F, C and R treatment for patients ≤60 years old (87.5 vs. 83.9%; P=0.24; Table II). For patients >60 years old, the ORR was slightly higher with F and C treatment compared with F, C and R treatment, however, there was no difference in OS irrespective of the administration of R (Figs. 8 and 9). In multivariate analysis, 17p^−^ and treatments without R were independent prognostic factors for a worse OS in younger patients, with hazard ratios (HRs) of 3.23 (95% CI, 0.327–31.98) and 4.9 (95% CI, 0.79–31.3), respectively. Binet stage C and elevated β2-MG levels were independent prognostic factors for a worse OS in older patients, with a HR of 2.8 (95% CI, 1.4–10.2) and 1.14 (95% CI, 0.56–3.41), respectively (Table III). Notably, R-based treatment was able to overcome the poor prognosis associated with 17p^−^ and significantly increased the 5-year OS from 52 to 74% (P=0.007, Fig. 10).

## Discussion

CLL is a mature B-cell neoplasm characterized by the expansion of CD5^+^ small- to medium-sized lymphocytes in the peripheral blood and is accompanied by other common disorders, such as lymphadenopathy, splenomegaly and hepatomegaly (9). Although CLL mainly demonstrates indolent behavior, a number of patients experience an aggressive course and succumb to the disease within a few years of diagnosis.

Combination therapies, such as F and C, have been developed and chemoimmunotherapy (CIT), which combines purine nucleoside analogs with or without alkylating agents and anti-CD20 monoclonal antibodies, have been used in previous years. Several studies have confirmed that the anti-CD20 monoclonal antibody R sensitizes CLL cells to the apoptotic effects of F (3–5). In the present study, CIT increased the ORR in patients and also caused a longer OS than cytotoxic chemotherapy alone. Similar or higher CR rates have been reported with combinations of F with C (2,10) or R (11) or F with C and R (3,4). The OS rates of patients 10 years after R and F-based CIT was equivalent to those demonstrated in CLL patients from Western countries (12,13).

Rituximab is a chimeric monoclonal antibody directed against the CD20 antigen and has become the standard drug used with chemotherapy for treating various B-cell lymphomas. In CLL, a low expression of the CD20 antigen on leukemic cells and poor response rates to a standard dose of R led to the initial expectation that R may not generate sufficient clinical benefits in this disease (14). However, another study demonstrated that higher doses of R used alone improved response rates (15). In this study, we observed a high percentage of CD20^+^ tumors (188 in 202 cases) and high expression levels of CD20 on CLL cells, including 61/188 that expressed CD20(+) and 127/188 that expressed CD20(++). The expression of CD20 was similar to other B-cell neoplasms. The majority of patients in this cohort did not receive a dose-escalation of R as this expensive antibody is not covered by health insurance in China. However, the results in Fig. 3 show that a routine dose of R in combination with chemotherapy achieved high response rates and a long OS in patients with CLL and may also have additive or synergistic effects in patients with 17p^−^. There is a difference between Western and Chinese patients with CLL. These results suggest that the patients with 17p^−^ may benefit from R-based CIT, which is consistent with results from a previous study (16).

The addition of R to F-containing regimens significantly improves the OS in younger patients, confirming the importance of this agent in current first-line CLL regimens for treating younger patients. This study showed that after R-based CIT, younger patients gained a greater benefit for 5-year OS (59%) than older patients (50.6%). According to SEER data, the 5-year survival rate of patients <55 years old in the United States is 88% (17). However, when compared with their age-matched control population, the life expectancy of younger patients with CLL is significantly reduced. While a younger age alone should not be considered as a reason to initiate therapy, the therapeutic goal for a young patient requiring therapy should focus on improving survival by achieving CR. The importance of achieving CR is emphasized by the finding that a better quality of remission is associated with longer survival times and notably, there were 39 patients who received R-based CIT that experienced grade 3 and 4 infections, compared with only eight patients who had received other treatments (including Ch) experiencing such infections. This difference may be explained by different etiologies. Infections which occur during Ch treatment may be correlated with transient neutropenia, whereas R and F treatments may be associated with prolonged B- or T-cell depletion (18).

Several clinical and pathological factors, including age, Binet stage and β2-MG and LDH levels, have been associated with prognosis. Biologically, an unfavorable course may be predicted by a variety of parameters, including the IgVH mutation status (19), ZAP-70 expression (20), genomic aberrations identified by FISH and serological proliferation markers. In univariate analysis, age >60 years, 17p^−^ and elevated β2-MG levels were associated with a worse OS in this study. Serum β2-MG levels have shown a strong prognostic effect in previous studies (13) and we confirmed this in the older patients of this study. The patients with normal β2-MG levels have a higher OS at five years compared with patients who had higher β2-MG levels. The presence of 17p^−^ was shown to be a strong negative prognostic indicator of progression-free and overall survival (3). Patients with this deletion had a significantly shorter OS in our study, irrespective of the treatment administered (Table I). Therefore, assessment of β2-MG levels and cytogenetic changes before treatment may be assayed to provide data for predicting the outcome of a patient. The combination of age, 17p^−^ and β2-MG levels may be useful to classify these patients.

Our study had limitations, as it was a retrospective single center study and had a small sample size. Hence, there was a chance of selection bias. However, we used standard statistical techniques to perform comparisons and carried out univariate and multivariate analysis in a non-selected population that received a uniform diagnostic and therapeutic approach.

In conclusion, younger patients with CLL benefited from R treatment, implying that the age of a patient is important in their response to therapy and survival.

## Figures and Tables

**Table I:** Clinical characteristics of the patients with CLL.

Variable	CR+PR (%)	P-value^b^	Median OS, months (95% CI)	P-value^c^
Age, years^a^				
≤60	62.8	0.112	70 (61–78.9)	0.031
>60	74.2		61 (54.9–73)	
Gender				
Male	71.6	0.652	69 (60.6–79)	0.732
Female	67.8		64 (57.1–72.3)	
Binet stage at diagnosis				
A	73.6	0.398	77 (64.5–91.1)	0.732
B	65.8		65 (57.7–73.6)	
C	75.7		59 (53.3–65.9)	
ECOG				
0	68.9	0.858	67 (56–77.9)	0.536
1	70.4		61 (46.5–75.4)	
≥2	80.0		53 (27.3–78.6)	
LDH				
≤450 IU/l	73.5	0.176	70 (52.3–87.6)	0.055
Elevated	64.5		61 (46.1–75.8)	
Serum β2-MG				
Normal	74.3	0.132	72 (57–86.9)	0.028
Elevated	63.9		59 (42.8–76.1)	
CD38				
Positive	66.0	0.085	65 (58.6–73.2)	0.175
Negative	79.6		65 (58.3–70.8)	
ZAP-70				
Positive	67.2	0.088	58 (52–63.9)	0.723
Negative	83.3		67 (59.5–74.4)	
IgVH mutational status				
Mutation	72.7	0.714	70 (60.4–80)	0.085
No mutation	68.7		65 (58.2–72.3)	
Genomic aberrations				
Trisomy 12	66.7	0.761	61 (47.3–75)	0.351
11q^−^	54.5	0.749	37 (23.4–51)	0.057
17p^−^	40.0	0.008	33 (20.4–45.5)	0.001
13q^−^	33.3	0.150	49 (34.2–64.6)	0.939
F-based treatment				
Yes	77.8	0.082	85 (72.3–98.4)	0.062
No	65.2		79 (63.5–93)	
R-based treatment				
Yes	68.5	0.067	71 (68.4–73.5)	0.218
No	58.5		61 (54.6–67.3)	

a The median age was 60.2 years old (range, 35–92);

b Fisher’s exact test;

c log-rank test. An age of >60 years, 17p^−^ and elevated β2-MG were associated with a worse OS in univariate analysis. 17p^−^ and treatments without R were independent prognostic factors for a worse OS in younger patients in multivariate analysis. Binet stage C and elevated β2-MG were independent prognostic factors for worse OS in older patients. CLL, chronic lymphocytic leukemia; OS, overall survival; LDH, lactate dehydrogenase; β2-MG, β2-microglobulin; IgVH, immunoglobulin variable heavy-chain; 17p^−^, chromosome 17p deletion; F, fludarabine; R, rituximab.

**Table II:** ORR of the patients with different treatments.

Treatment	≤60 years			>60 years		
	
	n	ORR (%)	P-value^a^	n	ORR (%)	P-value^a^
FC	19	87.5	0.24	31	76.6	0.28
FCR	21	83.9		33	73.2	

a Fisher’s exact test. ORR, overall response rate (CR+PR); CR, complete remission; PR, partial remission; F, fludarabine; C, cyclophosphamide; R, rituximab. FC treatment produced a higher, but not significantly higher, ORR than FCR treatment for patients with chronic lymphocytic leukemia, irrespective of age.

**Table III:** Comparison of the clinical characteristics of CLL patients and their outcome association (OS) according to age.

Clinical characteristic	>60 years group			≤60 years group		
	
	5-year OS	HR (95% CI)^a^	P-value^b^	5-year OS	HR (95% CI)^a^	P-value^b^
Overall	0.506			0.59		
Binet Stage						
A+B	0.589	0.356 (0.098–0.667)	0.003	0.659	0.616 (0.196–1.93)	0.073
C	0.166	1		0.543	1	
LDH						
Elevated	0.5	10.97 (1.62–74.2)	0.114	0.489	3.19 (0.6–17.4)	0.064
Normal	0.591	1		0.651	1	
Serum β2-MG						
Elevated	0.476	1.14 (0.56–3.41)	0.012	0.44	1.685 (0.123–3.81)	0.051
Normal	0.57	1		0.601	1	
CD38						
Positive	0.501	2.51 (0.89–7.47)	0.438	0.52	1.477 (0.053–4.31)	0.51
Negative	0.574	1		0.622	1	
ZAP-70						
Positive	0.502	7.619 (1.018–57.04)	0.148	0.57	2.95 (0.04–7.61)	0.157
Negative	0.553	1		0.631	1	
IgVH mutation						
Yes	0.641	0.128 (0.029–0.57)	0.067	0.676	0.244 (0.032–1.87)	0.062
No	0.44	1		0.512	1	
FISH						
17p^−^	0 (NA)	2.34 (0.644–22.06)	0.06	0 (NA)	3.23 (0.327–31.98)	0.001
11q^−^	0.201	1.75 (0.53–7.88)	0.251	0.392	1.84 (0.49–7.61)	0.525
Others	0.59	1		0.642	1	
Treatment						
F-containing	0.591	0.02 (0.002–0.189)	0.091	0.622	0.274 (0.053–1.41)	0.123
R-containing	0.701	0.251 (0.038–1.66)	0.052	0.739	0.203 (0.032–1.266)	0.020
Others	0.501	1		0.494	1	

a HR is relative to the reference category (with HR = 1);

b P-value of the Wald test for the HR, indicating the signficance of the association between the change of each item and outcome. NA, not applicable. CLL, chronic lymphocytic leukemia; OS, overall survival; HR, hazard ratio; LDH, lactate dehydrogenase; β2-MG, β2-microglobulin; IgVH, immunoglobulin variable heavy-chain; FISH, flourescence *in situ* hybridization; 17p^−^, chromosome 17p deletion; F, fludarabine; R, rituximab.
